# Assessment of pan coefficient models for the estimation of the reference evapotranspiration in a Mediterranean environment in Turkey

**DOI:** 10.7717/peerj.13554

**Published:** 2022-06-08

**Authors:** Deniz Levent Koç

**Affiliations:** Agricultural Structures and Irrigation/Agriculture Faculty, Çukurova University, Adana, Turkey

**Keywords:** Evapotranspiration, FAO-56 Penman-Monteith (PM), Pan coefficient models, Mediterranean environment

## Abstract

Reference evapotranspiration (ETo) is essential for irrigation practices and the management of water resources and plays a vital role in agricultural and hydro-meteorological studies. The FAO-56 Penman-Monteith (PM) equation, recommended as the sole standard method of calculating ETo by the Food and Agriculture Organization of the United Nations (FAO), is the most commonly used and accurate model to determine the ETo and evaluate ETo equations. However, it requires many meteorological variables, often restricting its applicability in regions with poor or missing meteorological observations. Many empirical and semi-empirical equations have been developed to predict the ET_0_ from numerous meteorological data. The FAO-24 Pan method is commonly used worldwide to estimate ETo because it is simple and requires only pan coefficients. However, pan coefficients (K_pan_) should be determined accurately to estimate ET_0_ using the FAO-24 Pan method. As the accuracy and reliability of the K_pan_ models can be different from one location to another, they should be tested or calibrated for different climates and surrounding conditions. In this study, the performance of the eight K_pan_ models was evaluated using 22-year daily climate data for the summer growing season in Adana, which has a Mediterranean climate in Turkey. The results showed that the mean seasonal pan coefficients estimated by all K_pan_ models differed significantly at a 1% significance level from those observed by FAO-56 PM according to the two-tail z test. In the study, ETo values estimated by K_pan_ models were compared against those obtained by the FAO-56 PM equation. The seasonal and monthly performance of K_pan_ models was varied, and the Wahed & Snyder model presented the best performance for ETo estimates at the seasonal scale. (RMSE = 0.550 mm d^−1^; MAE = 0.425 mm d^−1^; MBE = −0.378 mm d^−1^; RE = 0.134). In addition, it showed a good performance in estimating ETo on a monthly scale. The Orang model showed the lowest performance in estimating ETo among all models, with a very high relative error on the seasonal scale. (RMSE = 1.867 mm d^−1^; MAE = 1.806 mm d^−1^; MBE = −1.806 mm d^−1^; RE = 0.455). In addition, it showed the poorest performance on a monthly scale. Hence, the Wahed & Snyder model can be considered to estimate ETo under Adana region conditions after doing the necessary calibration.

## Introduction

Adana is situated in the northeast of the Mediterranean region, and it is the gateway to the Cilicia plain. In Adana, Irrigation is essential in crop production because rainfall amounts and their distribution are inadequate during the crop season. The summer growing season for main crops is between April and October. Several crops can be planted in the same crop year in Adana, where fertile cultivated land occupies a large area due to the suitable climate. Cereal, fruit, vegetable, and citrus are grown in the region, and their production efficiency is high (Kafalı Yılmaz, 2019).

It is fundamental to determine the water demands of irrigated plants for effective water management. A simple way to determine crop water requirements is to calculate reference evapotranspiration (ETo). Accurate estimation of crop reference evapotranspiration (ETo) is essential in managing water resources efficiently and scheduling farm irrigation (Grismer et al., 2002). Reference evapotranspiration (ETo) is a vital numerical tool for scientific and management models and decision frameworks in arid and semi-arid regions. Specifically, in irrigated agriculture, reliable estimates of ETo are crucial for water management to enhance ecosystem conservation and increase water productivity (Negm, Minacapillia & Provenzanoa, 2018).

It is common to estimate plant water use and water demands using crop reference evapotranspiration (ETo) and appropriate crop coefficients (K_c_) in different agroclimatic regions. The Food and Agriculture Organization (FAO) of the United Nations recommended the FAO-56 Penman-Monteith (PM) equation as the standard for estimation of reference evapotranspiration (ETo) and evaluation of the ETo equations (Allen et al., 1998). The fundamental difficulty in using the FAO-56 PM equation is the requirement of adequate weather data, which may not be available in most meteorological stations. Furthermore, evapotranspiration estimation depends upon the quality of the meteorological data. Agrometeorological stations may not be equipped to run this model (Chiew et al., 1995). For this reason, the FAO-24 Pan equation is being used commonly in irrigation projects and estimating ETo (Irmak, Haman & Jones, 2002).

The pan coefficient should be determined, which depends on upwind fetch distance, wind speed, and relative humidity, to estimate ETo using the FAO-24 Pan equation (Grismer et al., 2002). Doorenbos & Pruitt (1977) suggested a table for a few fetch distances under different relative humidity and wind speed to estimate the value of K_pan_. For a more accurate estimation of ETo, several equations have been further developed to determine the pan coefficient, and many studies have been conducted to convert pan evaporation to ETo. The researchers investigated and compared different pan coefficient models concerning their suitability for ETo estimation. Irmak, Haman & Jones (2002) evaluated two K_pan_ models using 23-year climate data in north-central Florida. They found an overestimation of ETo obtained by the Snyder model compared with the FAO-56 PM method, whereas ETo values obtained by the Frevert model performed well. In a study in a semi-arid region in Brazil conducted by Sentelhas & Folegatti (2003), the Pereira and Cuenca models made the best ETo estimates when compared ETo measured with the automatic weighing lysimeter. In contrast, Snyder was the worst model with the highest error among the K_pan_ models. In another study performed in a semi-arid region in India, Gundekar, Khodke & Sarkar (2008) estimated ETo values using pan coefficient models. They compared them with ETo values calculated by the FAO-56 PM method. According to the results, the Snyder K_pan_ model was the best suited for this region, whereas the Pereira, Cuenca, and Orang K_pan_ models gave a poor performance. Sabziparvar et al. (2010) evaluated seven K_pan_ models in two different climate regions of Iran. Orang and Raghuwanshi & Wallender were the best K_pan_ models under cold semi-arid climate conditions, whereas Snyder and Orang models had the best performance under warm, arid climate conditions. Rahimikhoob (2009) evaluated four K_pan_ models using 10-year mean climate data for a subtropical climate in the north of Iran. The results showed that the Orang model gave more accurate ETo estimates than other K_pan_ models. Cuenca, Snyder models significantly overestimated ETo compared with the FAO-56 PM method. In another study performed in an arid region in Iran, Heydari & Heydari (2014) used 21 years of climate data. They determined the Cuenca model showed the best adaptation compared with the FAO-56 PM. Wahed & Snyder and Raghuwanshi & Wallender models were successful in predicting ETo. Aschonitis, Antonopoulos & Papamichail (2012) evaluated six K_pan_ models in the Thessaloniki region in Greece. They found that the Cuenca model indicated the best adaptation to the ASCE-PM method compared to the other models. Khobragade et al. (2019) developed pan coefficients for a humid tropical monsoon region, Chandigarh in India, using the optimization technique. Researchers found a value of 0.92 as an optimized pan coefficient for the annual time scale, and the pan coefficients varied significantly by month and season for the study area. In addition, most of the popular K_pan_ models are unsuitable for the study area. Singh et al. (2014) performed a study in a semi-arid region in India. The results showed that ETo calculated from Modified Snyder and Orang model agreed with the FAO-56 PM method for daily, monthly, and annual estimates compared to other approaches. Another study performed by Pradhan et al. (2013) in a semi-arid region in India indicated that the Snyder model gave close agreement with the FAO-56 PM method, followed by the Cuenca, Orang, and Allen & Pruitt models. According to study results performed in a dry sub-humid region in India by Kar et al. (2017), the Snyder model indicated close agreement with the FAO-56 PM method. The values estimated by the Pereira model were mismatched with the FAO-56 PM method. In northern Iran’s mild, humid climate, Tabari, Grismer & Trajkovic (2013) evaluated eight K_pan_ models with the FAO-56 PM method. The ETo estimates computed by the Snyder model best matched the ETo estimates by the FAO-56 PM method, whereas the Pereira model gave the greatest underestimate of the ETo.

Considering the FAO-56 PM, this method is recommended as the sole standard approach for estimating ETo and validating other models (Allen et al., 1998). This method can be applied in various environments and climate conditions without local calibration and has been validated using lysimeters under a wide range of climatic conditions (Landeras, Ortiz-Barredo & Lo’pez, 2008; Shiri et al., 2012; Feng et al., 2017). But the main limitation of this method is that it requires many meteorological inputs that are not commonly available, especially in developing countries (Feng et al., 2017). Thus, the FAO-24 Pan method is widely used to estimate ETo because it is simple and requires daily Class A pan evaporation and pan coefficients (Irmak, Haman & Jones, 2002). There are only a few reported studies (Aschonitis, Antonopoulos & Papamichail, 2012; Babakos et al., 2020; Grismer et al., 2002) in which popular pan coefficient models were evaluated under Mediterranean climate conditions. Therefore, the present study aimed to assess the performances of K_pan_ models for reference evapotranspiration estimation based on pan evaporation conversions. The ETo obtained by eight popular K_pan_ models using the 22-year daily climate data were compared to the ETo obtained by the FAO-56 PM model in Adana, which has a hot-summer Mediterranean climate.

## Materials & Methods

### Study area

**Figure 1:**
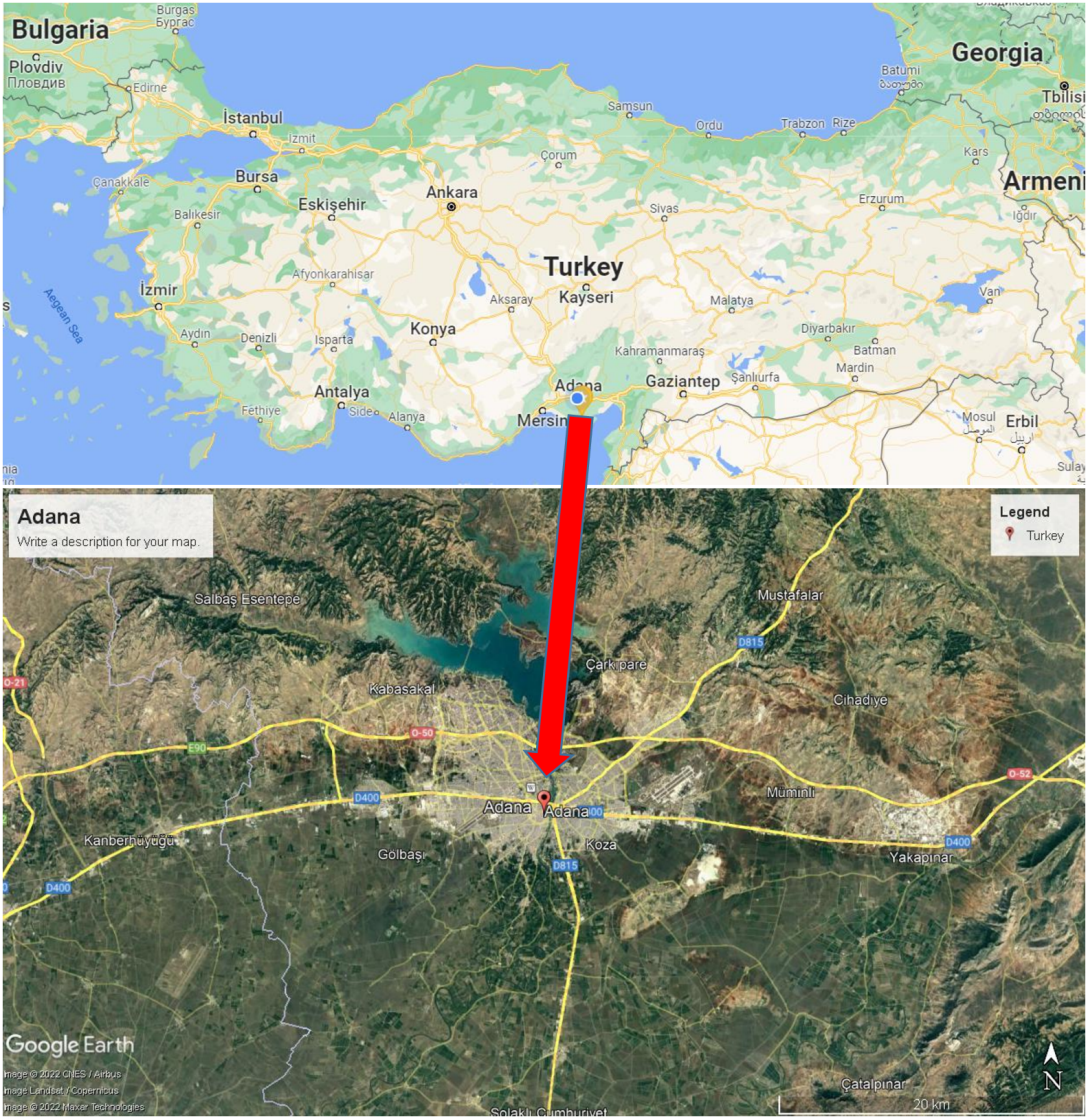
Location of the Adana in the Turkey map (Maps data: Google, CNES/Airbus, Landset/Copernicus, Maxar Technologies).

In the present study, the Adana weather station (latitude = 37°00′14″N; longitude = 35°20′39′E) located in central Adana operated by the Turkish State Meteorological Service (TSMS) is used to assess pan coefficient models. Adana plain is Turkey’s most extensive and fertile delta plain (Fig. 1). It consists of two parts called Çukurova and Upper Plain (Kafalı Yılmaz, 2019). Adana has a hot-summer Mediterranean climate. The study area is on flat terrain at an altitude of 24 m above sea level. According to Köppen–Geiger classification, Adana is Csa (warm temperate, summer dry, hot summer) (Öztürk, Çetinkaya & Aydın, 2017). According to the long-term measurement period (1929–2020), the rain in Adana falls mainly in the winter. The average annual rainfall is 668.1 mm, of which about 50% of rainfall occurs in December, January, and February, the three winter months. The months with the least precipitation are July and August, with an average of 10.2 and 9.6 mm, respectively. June and August are the hottest months, with a mean daily temperature of 28.2 and 28.7 °C, whereas January and February are the coldest months, with a mean daily temperature of 9.5 and 10.5 °C, respectively (Turkish State Meteorological Service, 2021). According to the long-term measurement period (1990-2019), the daily mean relative humidity is 65.8% in winter months while it is 68.9% in summer months, and its annual average is 66.0%. In the same measurement period, the daily mean wind speed is 1.33 in the winter months, whereas it is 1.40 in the summer months, and its annual average is 1.31 m s^−1^ (Turkish State Meteorological Service, 2020).

### Models and approaches

Eight pan coefficients (K_pan_) models derived from some weather data were tested and summarized below in Table 1. Long-term daily climatic parameters from 1998 to 2019 were used for this study.

**Table 1:** Pan coefficient models used in the study.

Model	References	Formula
Cuenca	Cuenca (1989)	K_pan_ = 0.475 − (2.4 × 10^−4^× U) + (5.16 × 10^−3^× RH) + (1.18 × 10^−3^× F) − (1.6 × 10^−5^× RH^2^) − (1.01 × 10^−6^× F^2^) − (8 × 10^−9^× RH^2^× U) − (1 × 10^−8^× RH^2^× F)
Allen and Pruitt	Allen & Pruitt (1991)	K_pan_ = 0.108 − 0.000331 × U + 0.0422 × ln(F) + 0.1434 × ln(RH) − 0.000631 × [ln(F)]^2^× ln(RH)
Snyder	Snyder (1992)	K_pan_ = 0.482 + 0. 024 × ln(F) –0.000376 × U + 0.0045 × RH
Pereira	Pereira et al. (1995)	K_pan_ = 0.85× (Δ + *γ*)/[Δ + *γ*× (1 + $ \frac{{r}_{c}}{{r}_{a}} $)] $\Delta = \frac{4098~\times ~ \left[ 0.6108~\times \exp ( \frac{17.27\times T}{T+237.3} ) \right] }{(T+237.3)^{2}} $
Orang	Orang (1998)	K_pan_ = 0.512062 − 0.000321 × U + 0.002889 × RH + 0.031886 × ln(F) − 0.000107 × RH × ln(F)
Raghuwanshi and Wallender	Raghuwanshi & Wallender (1998)	K_pan_ = 0.5944 + 0.0242 × X1 − 0.0583 × X2 − 0.1333 × X3 − 0.2083 × X4 + 0.0812 × X5 + 0.1344 × X6(X1 = ln(F), X2, X3, X4 = 0 if U < 175, X2 = 1 if 175 ≤ U < 425, X3 = 1 if 425 ≤ U < 700, X4 = 1 if U > 700 (km day^−1^), X5, X6 = 0 if RH < 40%, X5 = 1 if 40% ≤ RH < 70%, X6 = 1 if RH ≥ 70%)
Modified Snyder	Grismer et al. (2002)	K_pan_ = 0.5321 − 0.00030 × U + 0.0249 × ln(F) + 0.0025 × RH
Wahed and Snyder	Wahed & Snyder (2008)	K_pan_ = 0.62407 − 0.0266 × ln(F) − 0.00028 × U + 0.00226 × RH

**Notes.**

where U = mean daily wind speed at 2 m height (km day^−1^); F = the upwind fetch distance of low-growing vegetation (m) (Fetch was taken 50 m due to location of the Class A pan); RH = mean daily relative humidity (%); Δ = slope of saturation water vapor pressure curve at daily mean temperature (kPa K^−1^), T = mean daily air temperature (^o^C); *γ* = psychrometric constant (kPa K^−1^) (Psychrometric constant was calculated to be 0.06717 for the study area); r_c_ and r_a_ = canopy and aerodynamic resistance with r_c_ / r_a_ = 0.34 × U_2_ (Allen et al., 1998). U_2_ = mean daily wind speed at 2 m height (m s^−1^).

FAO-56 PM method was used to test the accuracy of the ETo estimated by K_pan_ models because FAO recommended it for different climatic conditions (Allen et al., 1998).

(1) \begin{eqnarray*}\mathrm{ETo}= \frac{0.408\times \Delta \times ({\mathrm{R}}_{\mathrm{n}}-\mathrm{G})+\gamma \times \frac{900}{\mathrm{T}+273} \times {\mathrm{U}}_{2}\times ({\mathrm{e}}_{\mathrm{s}}-{\mathrm{e}}_{\mathrm{a}})}{\Delta +\gamma \times (1+0.34\times {\mathrm{U}}_{2})} \end{eqnarray*}

where ETo = reference evapotranspiration (mm d^−1^); Δ = slope of vapor pressure curve (kPa.° C^−1^); R_n_ = mean daily net radiation (MJ m^−2^ d^−1^); G = mean daily soil heat flux density (MJ m^−2^ d^−1^); *γ* = psychrometric constant (kPa K^−1^); T = mean daily air temperature at 2 m height (°C); U_2_ = mean daily wind speed at 2 m height (m s^−1^); e_s_ = saturation vapor pressure (kPa); and e_a_ = actual vapor pressure (kPa); (e_s_ – e_a_) = saturation vapor pressure deficit (kPa) (Allen et al., 1998).

In this study, the ETo software (IAM_ETo) developed by Steduto & Snyder (1998) was used for determining the FAO-56 PM reference evapotranspiration. All of the data processing and calculations were performed in Microsoft Excel 2016.

Based on the FAO-24 Pan equation developed by Doorenbos & Pruitt (1977), pan evaporation data were converted to ETo using Eq. (2).

(2) \begin{eqnarray*}ETo={K}_{\mathrm{pan}}\times {E}_{\mathrm{pan}}\end{eqnarray*}

where K_pan_ = pan coefficient; E_pan_ = pan evaporation (mm d^−1^).

The ‘observed’ Class A pan coefficients were calculated by dividing FAO-56 PM ETo values by E_pan_ values (Eq. (3)).

(3) \begin{eqnarray*}{K}_{pan-obs.}= \frac{ETo}{{E}_{pan}} .\end{eqnarray*}

### Statistical analysis

A two-tail Z test was conducted to determine if the observed and models-estimated seasonal mean K_pan_ values differed significantly. Daily mean K_pan_ values observed and estimated during April-October in the years 1998–2019 were used in the Z-test. In addition, the five statistical parameters suggested by Karunanithi et al. (1994) and Jacovides & Kontoyiannis (1995) were used in the evaluation of the pan coefficient models in estimating ETo (Table 2). These five indices were calculated at the monthly and seasonal scales.

**Table 2:** Statistical parameters used to assess pan coefficient models.

Statistical parameter	Symbol	Equation
Root mean square error	RMSE	$RMSE=\sqrt{ \frac{1}{n} {\mathop{\sum }\nolimits }_{i=1}^{n}(ET{o}_{estimated,i}-ET{o}_{observed,i})^{2}}$
Relative error	RE	$RE= \frac{RMSE}{\overline{O}} $
Mean bias error	MBE	$MBE= \frac{1}{n} {\mathop{\sum }\nolimits }_{i=1}^{n}(ET{o}_{estimated,i}-ET{o}_{observed,i})$
Mean absolute error	MAE	$MAE= \frac{1}{n} {\mathop{\sum }\nolimits }_{i=1}^{n} \left\vert ET{o}_{estimated,i}-ET{o}_{observed,i} \right\vert $
t-statistic	t	$t= \left[ \frac{(n-1)MB{E}^{2}}{RMS{E}^{2}-MB{E}^{2}} \right] ^{1/2}$

**Notes.**

n = the total number of data; ETo_estimated_ = K_pan_ model-estimated ETo values; ETo_observed_ = FAO-56 PM ETo values; $\overline{O}$ = the mean of FAO-56 PM ETo values.

## Results and Discussion

### Estimation of pan coefficients

This study calculated daily and monthly mean K_pan_ values using eight K_pan_ models. The pan coefficients obtained using K_pan_ models and the FAO-56 PM equation are presented in Table 3. The pan coefficient values estimated using K_pan_ models showed a slight change during the season for each month, while those observed by FAO-56 PM showed variation among the months. It can be seen in Table 3 that K_pan_ values estimated by the Snyder model were higher than those of other models in all months during the season, while the Orang model gave lower pan coefficient values. The mean monthly pan coefficients during the season estimated by K_pan_ models ranged from 0.38 to 0.85, and those observed by FAO-56 PM ranged from 0.68 to 0.87. Allen & Pruitt (1991) and Babakos et al. (2020) reported that the range of pan coefficient values is nearly between 0.35 to 1.1 for various pan types and various local conditions of climates and surrounding environments.

**Table 3:** Mean monthly values of the observed K_pan-obs._ (*ETo/E_pan_) and computed K_pan_ by using K_pan_ models in years 1998–2019.

Month	^*^ETo/E_pan_	S	MS	WS	C	AP	O	P	RW
**April**	0.87	0.83	0.76	0.64	0.77	0.80	0.40	0.77	0.78
**May**	0.80	0.84	0.77	0.64	0.77	0.80	0.40	0.78	0.78
**June**	0.73	0.84	0.77	0.65	0.78	0.80	0.39	0.78	0.79
**July**	0.70	0.85	0.78	0.65	0.78	0.81	0.38	0.79	0.79
**August**	0.68	0.85	0.77	0.65	0.78	0.81	0.39	0.80	0.79
**September**	0.69	0.83	0.76	0.64	0.77	0.80	0.40	0.79	0.78
**October**	0.71	0.81	0.75	0.63	0.75	0.79	0.42	0.79	0.77
**Average**	0.74	0.84	0.77	0.64	0.77	0.80	0.40	0.79	0.78

**Notes.**

* ETo is calculated using FAO-56 PM equation.

S Snyder MS Modified Snyder WS Wahed & Snyder C Cuenca AP Allen & Pruitt O Orang P Pereira RW Raghuwanshi & Wallender

As shown in Table 3, observed K_pan_ values were lower in dry months (July, August, September), whereas they were higher (April and May) in the rainy months. Similar results have been found in the studies performed in a Mediterranean environment by Aschonitis, Antonopoulos & Papamichail (2012) and in a humid tropical climate by Pradhan et al. (2013). Aschonitis, Antonopoulos & Papamichail (2012) assessed six K_pan_ models by two years of E_pan_ measurements using ASCE-PM as a reference in the Thessaloniki plain in Greece, which has a semi-arid Mediterranean environment. They likewise determined that observed K_pan_ values were higher in rainy months (April and May) and lower in dry months (June, July, August). The mean monthly K_pan_ values estimated by K_pan_ models ranged from 0.32 to 0.75, and those observed by ASCE-PM ranged from 0.67 to 0.76.

The pan coefficient depends on wind speed (U), relative humidity (RH), and upwind fetch distance.

As shown in Table 3, observed K_pan_ values were 0.87 and 0.80 in April and May, respectively, whereas they ranged from 0.68 to 0.73 from June to October. However, K_pan_ coefficients estimated by models indicated a slight change in all months because RH and U values changed slightly during the season. The Snyder model estimated close to observed K_pan_ values in April and May, whereas the Wahed & Snyder model estimated close to observed K_pan_ values in August. In contrast, the Modified Snyder, Cuenca, Allen & Pruitt, Pereira, and Raghuwanshi & Wallender models estimated K_pan_ values close to observed K_pan_ values in May.

According to Z-test, the results showed that mean seasonal pan coefficients estimated by all K_pan_ models during April-October in the years 1998–2019 differed significantly at a 1% significance level from those observed by FAO-56 PM (*p* < 0.01, *n* = 214). The results were given in Table S1.

### Estimation of reference evapotranspiration

The daily Class A pan evaporation data and pan coefficients determined by K_pan_ models were used to estimate reference evapotranspiration (ETo). The ETo values estimated by eight K_pan_ models were tested against those calculated using the FAO-56 PM equation.

**Figure 2:**
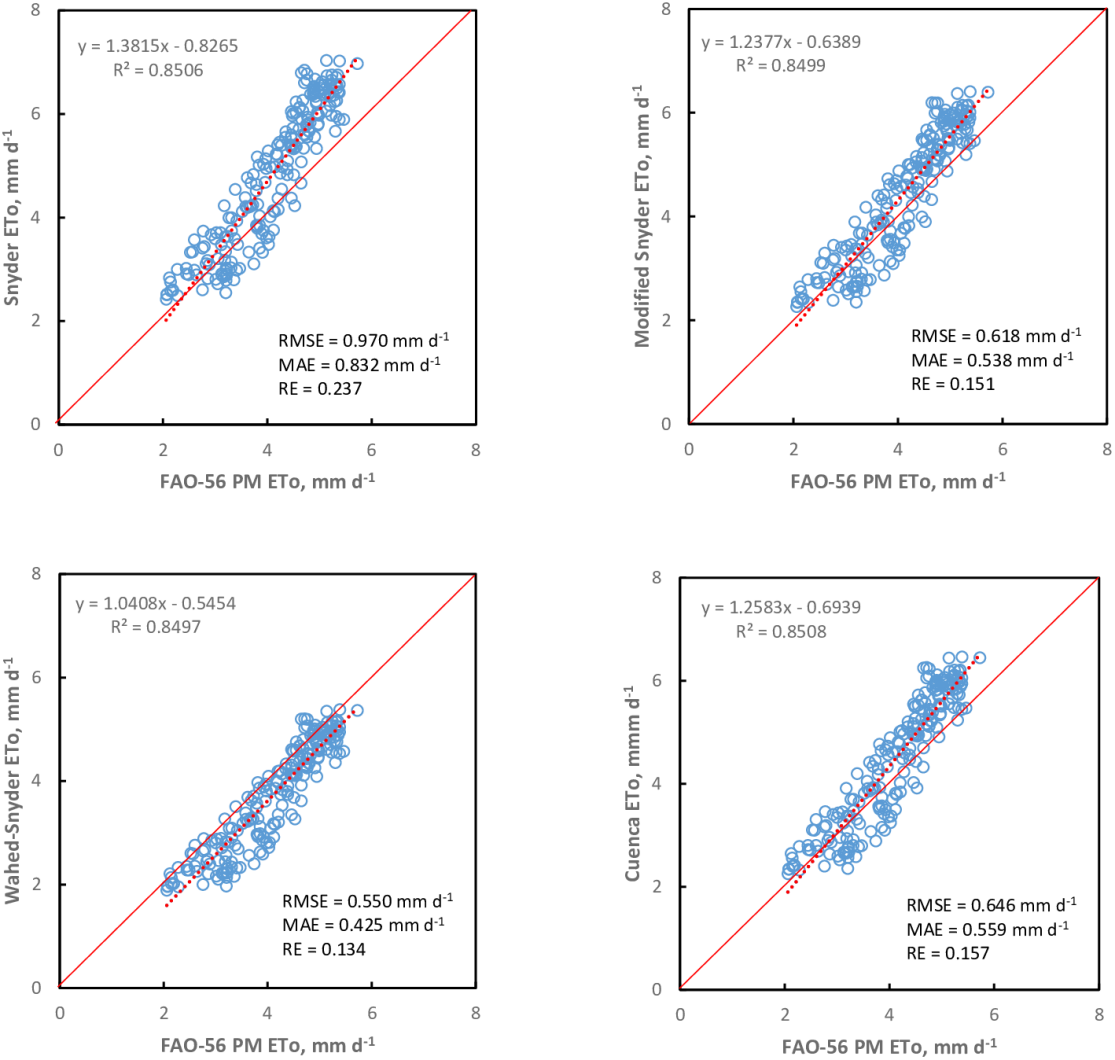
Performance of K_pan_ models estimating ETo at seasonal scale in the years 1998–2019. Each point represents 22 years of daily mean ETo (April to October).

**Figure 3:**
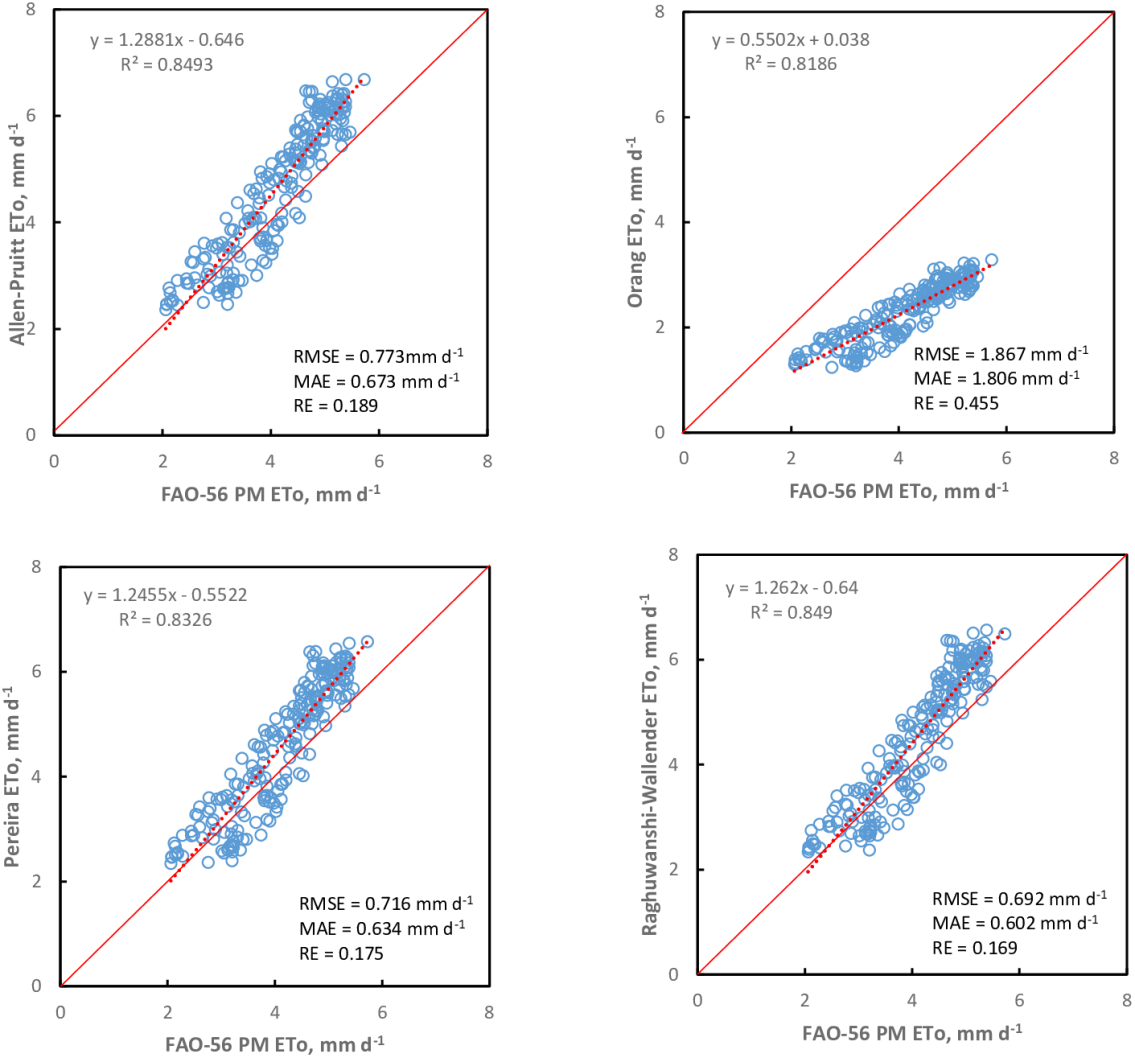
Performance of K_pan_ models estimating ETo at seasonal scale in the years 1998–2019. Each point represents 22 years of daily mean ETo (April to October).

The performance of the K_pan_ models according to RMSE, MAE, and RE statistics for seasonal scale is shown in Figs. 2 and 3. It can be seen from the scatter plot that the performance of the K_pan_ models varied for the seasonal scale. None of the K_pan_ models could estimate the ETo satisfactorily, but the Wahed & Snyder model had better performance to estimate ETo for the seasonal scale than other models (RMSE = 0.550 mm d^−1^; MAE = 0.425 mm d^−1^; RE = 0.134). The Wahed & Snyder model tended to underestimate ETo values during the season. In contrast, the Snyder, Modified Snyder, Cuenca, Allen & Pruitt, Pereira, and Raghuwanshi & Wallender models often overestimated ETo values. The Orang K_pan_ model underestimated ETo values during the season. It can be seen in Fig. 3 the Orang model resulted in the highest relative error at the seasonal scale [RE (%) = 45.5] compared to other models. According to the results, the accuracy of the K_pan_ models was as follows ranking. Wahed & Snyder >Modified Snyder >Cuenca >Raghuwanshi & Wallender >Pereira >Allen & Pruitt >Snyder >Orang.

Similarly, Aschonitis, Antonopoulos & Papamichail (2012) reported that Snyder and Orang were the worst models to estimate ETo in a semi-arid Mediterranean environment in Thessaloniki in Greece. However, they found that the Cuenca model indicated the best adaptation to the ASCE PM equation compared to the other K_pan_ models. Grismer et al. (2002) concluded that among the six K_pan_ equations and FAO-24 table, the Allen and Pruitt K_pan_ model yielded better ETo estimations than other K_pan_ models in California conditions with a Mediterranean climate. Cobaner (2013) used three pan-based equations: the FAO-24 Pan (Doorenbos & Pruitt, 1977), the Snyder ETo (Snyder et al., 2005), and the Ghare ETo (Ghare, Porey & Ingle, 2006) in the Fresno and Bakersfield weather stations located in California, which has a Mediterranean climate. In his study, the daily Class A pan evaporation and reference evapotranspiration data are used as inputs to the wavelet regression (WR) models to estimate the ETo obtained using the FAO-56 PM equation. The performance of the WR models is compared with those of empirical models and their calibrated versions. His study selected the Allen & Pruitt K_pan_ model for the FAO-24 Pan equation because Grismer et al. (2002) determined it as the best K_pan_ model in California conditions. The WR model and the FAO-24 Pan equation yielded more accurate ETo estimation than those of the Snyder ETo and Ghare ETo equations. The WR model performed slightly better in predicting the ETo than the FAO-24 Pan equation. Trajkovic & Kolakovic (2010) evaluated the reliability of pan-based approaches (FAO-24 Pan, Snyder ETo, Ghare ETo) for estimating ETo, comparing them against daily lysimeter data from Policoro, Italy, which has a semi-arid Mediterranean climate. K_pan_ values obtained by radial basis function (RBF) were used in the study because Trajkovic & Kolakovic (2010) presented that the RBF network predicted K_pan_ values better than the K_pan_ equations of Frevert, Hill & Braaten (1983) and Snyder (1992). The results indicated that the Snyder equation estimated the ETo better than the FAO-24 Pan equation, although it does not require the relative humidity and wind speed data. Estimating the pan coefficients with the RGF model but not determining the most appropriate pan coefficient model for the region may have caused this result. In a study conducted by Gundekar, Khodke & Sarkar (2008) in semi-arid conditions in India, Pereira, Cuenca, and Orang K_pan_ models showed the worst performance to estimate ETo. However, the Snyder K_pan_ model showed the best performance to estimate ETo in the study. Sabziparvar et al. (2010) also reported that the Snyder K_pan_ model gave the best performance for the warm-arid climate of Iran. On the other hand, it gave a poor performance in a study performed by Sentelhas & Folegatti (2003) in a warm-humid environment in Brazil and another study in the humid tropical region of India by George (2012).

It can be concluded that the pan coefficient is very dependent on local and climatic conditions and should be determined by comparing the pan data with the FAO-56 PM ETo estimates (Chiew et al., 1995).

The values of RMSE, MBE, RE, MAE, and t-statistic used to evaluate the K_pan_ models for the monthly scale are given in Tables 4–8. The results in Tables 4–8 show that the Wahed & Snyder model performed better than other models, mainly from June to October. However, it gave poor performance in the rainy months (April, May). The Wahed & Snyder model had the smallest RMSE, RE, MAE, MBE, and t values from June to October among the models. All models showed varied relative errors (RE) by months (Table 6). According to models, RE as a percentage ranges from 5.5% to 54.4%. The Wahed & Snyder model errors ranged from 5.5% to 10.1% from June to October. The highest error happened in April at 28.4%. The Orang model showed the highest error, ranging from 37.2% to 54.4% from April to October (Table 6). The errors shown by other models during April-October were as follows: Snyder (10.3%–31%), Modified Snyder (9.7%–19.8%), Cuenca (9.8%–20.8%), Allen & Pruitt (10.2%–24.9%), Pereira (9.7%–23.1%), Raghuwanshi & Wallender (10.0%–22.3%).

As shown in Table 5, according to MBE values, K_pan_ models overestimated and underestimated mean ETo values according to months. Snyder and Raghuwanshi & Wallender models underestimated ETo values in April, but they overestimated ETo in other months. The Modified Snyder, Cuenca, and Pereira models underestimated ETo values in April and May. In contrast, these models overestimated ETo values in other months. Wahed & Snyder and Orang models underestimated ETo in all months.

**Table 4:** RMSE values for monthly mean ETo (mm d^−1^) estimated by K_pan_ models in years 1998–2019.

Month/Model	April	May	June	July	August	September	October
**S**	0.350	0.515	1.004	1.358	1.449	0.951	0.559
**MS**	0.526	0.405	0.536	0.782	0.922	0.586	0.364
**WS**	0.963	0.816	0.504	0.314	0.258	0.233	0.247
**C**	0.515	0.406	0.572	0.840	0.971	0.609	0.364
**AP**	0.418	0.426	0.750	1.028	1.160	0.780	0.487
**O**	1.844	2.030	2.225	2.276	1.898	1.489	0.958
**P**	0.505	0.403	0.638	0.887	1.076	0.754	0.495
**RW**	0.472	0.415	0.652	0.903	1.040	0.661	0.429

**Table 5:** MBE values for monthly mean ETo (mm d^−1^) estimated by K_pan_ models in years 1998–2019.

Month/Model	April	May	June	July	August	September	October
**S**	−0.217	0.260	0.945	1.327	1.407	0.912	0.512
**MS**	−0.464	−0.094	0.441	0.737	0.869	0.541	0.303
**WS**	−0.937	−0.750	−0.430	−0.208	−0.018	−0.153	−0.164
**C**	−0.450	−0.072	0.481	0.797	0.920	0.563	0.300
**AP**	−0.329	0.092	0.679	0.992	1.116	0.741	0.439
**O**	−1.835	−2.019	−2.216	−2.269	−1.891	−1.478	−0.938
**P**	−0.438	−0.004	0.559	0.846	1.032	0.716	0.453
**RW**	−0.394	0.005	0.568	0.862	0.991	0.616	0.375

**Table 6:** RE values for monthly mean ETo (mm d^−1^) estimated by K_pan_ models in years 1998–2019.

Month/Model	April	May	June	July	August	September	October
**S**	0.103	0.124	0.187	0.263	0.310	0.253	0.217
**MS**	0.155	0.097	0.108	0.152	0.198	0.156	0.141
**WS**	0.284	0.196	0.101	0.061	0.055	0.062	0.096
**C**	0.152	0.098	0.115	0.163	0.208	0.162	0.141
**AP**	0.123	0.102	0.151	0.199	0.249	0.207	0.189
**O**	0.544	0.488	0.448	0.441	0.407	0.396	0.372
**P**	0.149	0.097	0.128	0.172	0.231	0.200	0.192
**RW**	0.139	0.100	0.131	0.175	0.223	0.175	0.166

In this study, monthly mean ETo values estimated by K_pan_ models ranged from 1.55 to 6.49 mm d^−1^, whereas observed monthly mean ETo values ranged from 2.58 to 5.16 mm d^−1^ (Table 9). The Wahed & Snyder model estimated the lowest monthly mean ETo with 2.41 mm d^−1^ in October and the highest monthly mean ETo with 4.95 mm d^−1^ in July. The Orang and Snyder models produced the least accurate monthly mean ETo estimates. The Wahed & Snyder model accurately estimated the monthly mean ETo in August. In addition, it showed good performance estimating ETo values in July, September, and October with small t values (4.705−4.870), as seen in Table 8. The Wahed & Snyder model had the smallest t values from July to October, whereas the Orang model had the highest t values during the season. Modified Snyder, Cueanca, Allen & Pruitt, Pereira, and Raghuwanshi & Wallender models accurately estimated the mean monthly ETo in May. Similarly, Irmak, Haman & Jones (2002), Gundekar, Khodke & Sarkar (2008), and Mahmud et al. (2020) indicated the success of K_pan_ models in accurately predicting ETo varied according to months.

**Table 7:** MAE values for monthly mean ETo (mm d^−1^) estimated by K_pan_ models in years 1998–2019.

Month/Model	April	May	June	July	August	September	October
**S**	0.301	0.405	0.945	1.327	1.407	0.912	0.514
**MS**	0.476	0.357	0.460	0.737	0.869	0.541	0.318
**WS**	0.937	0.750	0.447	0.247	0.209	0.192	0.200
**C**	0.463	0.360	0.492	0.797	0.920	0.563	0.316
**AP**	0.362	0.370	0.679	0.992	1.116	0.741	0.444
**O**	1.835	2.019	2.216	2.269	1.891	1.478	0.938
**P**	0.454	0.363	0.562	0.846	1.032	0.716	0.456
**RW**	0.412	0.367	0.572	0.862	0.991	0.616	0.386

**Table 8:** t values for monthly mean ETo (mm d^−1^) estimated by K_pan_ models in years 1998–2019.

Month/Model	April	May	June	July	August	September	October
**S**	4.259	3.192	15.009	25.404	22.202	18.031	12.463
**MS**	10.104	1.308	7.794	15.438	15.491	12.849	8.247
**WS**	22.770	12.778	8.797	4.855	0.385	4.705	4.870
**C**	9.649	0.990	8.360	16.520	16.176	13.054	8.018
**AP**	6.886	1.206	11.484	20.255	19.211	16.359	11.419
**O**	53.331	53.440	61.007	67.465	64.196	42.544	26.618
**P**	9.384	0.053	9.787	17.420	18.564	16.434	12.395
**RW**	8.138	0.070	9.542	17.582	17.151	13.838	9.920

**Notes.**

t_crit_ (0.01) = 2.750 (*n* = 31) and 2.756 (*n* = 30); t_crit_ (0.05) = 2.042 (*n* = 31) and 2.045 (*n* = 30).

**Table 9:** Mean monthly values of ETo (mm d^−1^) obtained by K_pan_ models and FAO-56 PM equation in years 1998–2019.

Month/Model	April	May	June	July	August	September	October
**FAO-56 PM**	3.39	4.16	4.97	5.16	4.67	3.77	2.58
**S**	3.17^**^	4.42^**^	5.92^**^	6.49^**^	6.07^**^	4.68^**^	3.09^**^
**MS**	2.92^**^	**4.07**	5.41^**^	5.90^**^	5.54^**^	4.31^**^	2.88^**^
**WS**	2.45^**^	3.41^**^	4.54^**^	4.95^**^	**4.65**	3.61^**^	2.41^**^
**C**	2.94^**^	**4.09**	5.45^**^	5.96^**^	5.59^**^	4.33^**^	2.88^**^
**AP**	3.06^**^	**4.25**	5.65^**^	6.15^**^	5.78^**^	4.51^**^	3.02^**^
**O**	1.55^**^	2.14^**^	2.76^**^	2.89^**^	2.78^**^	2.29^**^	1.64^**^
**P**	2.95^**^	**4.16**	5.53^**^	6.01^**^	5.70^**^	4.48^**^	3.03^**^
**RW**	3.00^**^	**4.17**	5.54^**^	6.02^**^	5.66^**^	4.38^**^	2.95^**^

**Notes.**

Mean monthly values which differed significantly from FAO-56 PM (reference method) according to t-statistic.

* *p*-value < 0.05.

** *p*-value < 0.01.

Significant values are indicated in bold.

## Conclusions

Eight K_pan_ models were assessed for their potential to estimate reference evapotranspiration. The FAO-56 PM equation was used as a reference to evaluate the K_pan_ models. Generally, the K_pan_ models showed inadequate performance in estimating ETo for monthly and seasonal scales. Orang model showed the poorest performance in estimating ETo monthly and seasonal scales. However, the Wahed & Snyder model showed the best performance in estimating ETo at monthly and seasonal scales among the models. The Wahed & Snyder model accurately estimated the monthly mean ETo in August. In addition, it showed good performance by estimating mean ETo from June to October with ranging of 5.5% to 10.1% relative errors. However, the Wahed & Snyder model still cannot be recommended as an alternative to the FAO-56 PM equation. There is a need for its calibration in estimating ETo in Adana region conditions.

##  Supplemental Information

10.7717/peerj.13554/supp-1 Supplemental Information 1 Z-Test: Two Sample for Means (Alpha =0.01)

10.7717/peerj.13554/supp-2 Supplemental Information 2 Raw data

## Additional Information and Declarations

### Data Availability

The following information was supplied regarding data availability: The raw measurements are available in the Supplementary File.
