# Supplementary material for: Assessment of pan coefficient models for the estimation of the reference evapotranspiration in a Mediterranean environment in Turkey
Source: PeerJ. 2022 Jun 8;10:e13554. doi: 10.7717/peerj.13554 (PMC9188310; doi:10.7717/peerj.13554)
Supplement: Supplemental Information 1 [file peerj-10-13554-s001.docx]

**Supplement Table1.** Z-Test: Two Sample for Means (Alpha=0.01)

|  | **Snyder** | **FAO56-PM** |
| --- | --- | --- |
| Mean | 0.836818776 | 0.740266278 |
| Known Variance | 0.000248 | 0.005545 |
| Observations | 214 | 214 |
| Hypothesized Mean Difference | 0 |  |
| **z** | 18.56227135 |  |
| P(Z<=z) one-tail | 0 |  |
| z Critical one-tail | 2.326347874 |  |
| P(Z<=z) two-tail | 0 |  |
| **z Critical two-tail** | 2.575829304 |  |

|  | **Modified Snyder** | **FAO56-PM** |
| --- | --- | --- |
| Mean | 0.767023242 | 0.740266278 |
| Known Variance | 0.000072 | 0.005545 |
| Observations | 214 | 214 |
| Hypothesized Mean Difference | 0 |  |
| **z** | 5.225914306 |  |
| P(Z<=z) one-tail | 8.66483E-08 |  |
| z Critical one-tail | 2.326347874 |  |
| P(Z<=z) two-tail | 1.73297E-07 |  |
| **z Critical two-tail** | 2.575829304 |  |

|  | **Wahed-Snyder** | **FAO56-PM** |
| --- | --- | --- |
| Mean | 0.643602606 | 0.740266278 |
| Known Variance | 0.000058 | 0.005545 |
| Observations | 214 | 214 |
| Hypothesized Mean Difference | 0 |  |
| **z** | -18.89627339 |  |
| P(Z<=z) one-tail | 0 |  |
| z Critical one-tail | 2.326347874 |  |
| P(Z<=z) two-tail | 0 |  |
| **z Critical two-tail** | 2.575829304 |  |

|  | **Cuenca** | **FAO56-PM** |
| --- | --- | --- |
| Mean | 0.772228984 | 0.740266278 |
| Known Variance | 0.00012 | 0.005545 |
| Observations | 214 | 214 |
| Hypothesized Mean Difference | 0 |  |
| **z** | 6.215015384 |  |
| P(Z<=z) one-tail | 2.56598E-10 |  |
| z Critical one-tail | 2.326347874 |  |
| P(Z<=z) two-tail | 5.13196E-10 |  |
| **z Critical two-tail** | 2.575829304 |  |

|  | **Allen-Pruitt** | **FAO56-PM** |
| --- | --- | --- |
| Mean | 0.801641592 | 0.740266278 |
| Known Variance | 0.000063 | 0.005545 |
| Observations | 214 | 214 |
| Hypothesized Mean Difference | 0 |  |
| **z** | 11.99793762 |  |
| P(Z<=z) one-tail | 0 |  |
| z Critical one-tail | 2.326347874 |  |
| P(Z<=z) two-tail | 0 |  |
| **z Critical two-tail** | 2.575829304 |  |

|  | **Orang** | **FAO56-PM** |
| --- | --- | --- |
| Mean | 0.396180461 | 0.740266278 |
| Known Variance | 0.000205 | 0.005545 |
| Observations | 214 | 214 |
| Hypothesized Mean Difference | 0 |  |
| **z** | -66.43817783 |  |
| P(Z<=z) one-tail | 0 |  |
| z Critical one-tail | 2.326347874 |  |
| P(Z<=z) two-tail | 0 |  |
| **z Critical two-tail** | 2.575829304 |  |

|  | **Pereira** | **FAO56-PM** |
| --- | --- | --- |
| Mean | 0.785278608 | 0.740266278 |
| Known Variance | 0.000111 | 0.005545 |
| Observations | 214 | 214 |
| Hypothesized Mean Difference | 0 |  |
| **z** | 8.760202621 |  |
| P(Z<=z) one-tail | 0 |  |
| z Critical one-tail | 2.326347874 |  |
| P(Z<=z) two-tail | 0 |  |
| **z Critical two-tail** | 2.575829304 |  |

|  | **Raghuwanshi-Wallender** | **FAO56-PM** |
| --- | --- | --- |
| Mean | 0.784053285 | 0.740266278 |
| Known Variance | 0.000113 | 0.005545 |
| Observations | 214 | 214 |
| Hypothesized Mean Difference | 0 |  |
| **z** | 8.5217329 |  |
| P(Z<=z) one-tail | 0 |  |
| z Critical one-tail | 2.326347874 |  |
| P(Z<=z) two-tail | 0 |  |
| **z Critical two-tail** | 2.575829304 |  |
